# Structure and Possible Functions of Constant-Frequency Calls in *Ariopsis seemanni* (Osteichthyes, Ariidae)

**DOI:** 10.1371/journal.pone.0064864

**Published:** 2013-05-31

**Authors:** Daniel Schmidtke, Jochen Schulz, Jörg Hartung, Karl-Heinz Esser

**Affiliations:** 1 Institute of Zoology, University of Veterinary Medicine, Hannover, Germany; 2 Center for Systems Neuroscience, University of Veterinary Medicine, Hannover, Germany; 3 Institute for Animal Hygiene, Animal Welfare and Farm Animal Behaviour, University of Veterinary Medicine, Hannover, Germany; Université Pierre et Marie Curie, France

## Abstract

In the 1970s, Tavolga conducted a series of experiments in which he found behavioral evidence that the vocalizations of the catfish species *Ariopsis felis* may play a role in a coarse form of echolocation. Based on his findings, he postulated a similar function for the calls of closely related catfish species. Here, we describe the physical characteristics of the predominant call-type of *Ariopsis seemanni*. In two behavioral experiments, we further explore whether *A. seemanni* uses these calls for acoustic obstacle detection by testing the hypothesis that the call-emission rate of individual fish should increase when subjects are confronted with novel objects, as it is known from other vertebrate species that use pulse-type signals to actively probe the environment. Audio-video monitoring of the fish under different obstacle conditions did not reveal a systematic increase in the number of emitted calls in the presence of novel objects or in dependence on the proximity between individual fish and different objects. These negative findings in combination with our current understanding of directional hearing in fishes (which is a prerequisite for acoustic obstacle detection) make it highly unlikely that *A. seemanni* uses its calls for acoustic obstacle detection. We argue that the calls are more likely to play a role in intra- or interspecific communication (e.g. in school formation or predator deterrence) and present results from a preliminary Y-maze experiment that are indicative for a positive phonotaxis of *A. seemanni* towards the calls of conspecifics.

## Introduction

Laryngeally echolocating bats and flying foxes of the genus *Rousettus* (both belonging to the Chiroptera), toothed whales (Odontoceti), and some cave-dwelling birds (i.e. species from the families Steatornithidae and Apodidae) use echolocation to determine their relative position to other objects in their environment by emitting calls and listening to the returning echoes [1]–[6]. Other rare examples of echolocation may exist in mammals including baleen whales, seals, shrews, and humans [7]–[11]. Echo information can be used for orientation, to reconstruct an image of the animals’ surroundings, or prey detection. When discussing echolocation, this ability is not usually attributed to bony fishes (Osteichthyes). However, previous studies done by William N. Tavolga [12], [13] have given reason to suspect that some bony fishes possess this ability in a more primitive form. Research on the Hardhead sea catfish (*Ariopsis felis*) was the first indicator that Osteichthyes might be able to use directional hearing for the acoustical detection of obstacles [13]. Observations correlated the emission of low-frequency sound pulses to the detection and avoidance of nearby obstacles as a coarse form of echolocation [12]. Single, intact animals within a group readily produced sound pulses and avoided barriers while muted animals frequently bumped into obstacles. Many other fishes are known to produce sounds, but their function has only been established in a few species. Fish sounds are typically associated with e.g. alarm, territorial defense, and courtship (pre-spawning) behavior [14]–[16] but have been related to sound probing of the environment only in *A. felis*.

In the present study, the Tete sea catfish (*Ariopsis seemanni),* a close relative of *A. felis* (for genetic distance see [17]), was selected for further investigation of the use of sound production for echolocation purposes in Osteichthyes. *A. seemanni* is common in coastal marine and brackish waters of Central and South America from Mexico to Peru [18]. *A. seemanni* seemed particularly suited for our follow-up study since, in this species, single fish vocalize virtually unceasingly even in the absence of conspecifics (D. Schmidtke and K.-H. Esser, unpublished observations). In contrast, individuals of *A. felis* were found to be less vocal and at least visual stimulation by other fish was required to induce normal sound production [12].

The majority of the sounds produced by different species of fishes are low-frequency pulses of short duration with major energy below 100 Hz. There are few species able to produce sounds at frequencies of 300 Hz and above [19]. If sound detection is considered, *A. felis*, as an example, is able to detect frequencies from 50 to 1,000 Hz, but is most sensitive at 100 to 200 Hz [20]. A few fish species (e.g. *Astyanax mexicanus*, *Astyanax jordani*; [21]) can detect sound frequencies of several kHz, while still others (a number of clupeid fishes) have more recently been found to even be able to sense acoustic signals in the ultrasonic range (up to at least 180 kHz; for review see [22]). However, in most hearing specialists, including the aforementioned *Ariopsis* species, the lowest auditory thresholds of about 50 to 60 dB (re 1 mPa) were found between 200 and 1,500 Hz and useful hearing ranges extended from below 100 to 2,000 Hz [23].

The first aim of the current study was to describe the physical properties of the predominant call type of the species *Ariopsis seemanni* in terms of duration and spectral composition and the dependence of the latter on environmental factors (size of the experimental environment and water temperature) and body length of the fish. The second aim of the study was to investigate if sound production of *A. seemanni* is related to a coarse form of echolocation, similar to what was previously suggested for *A. felis*, or if there is a different functional role of the calls.

To determine the possible function of the calls, three behavioral experiments were conducted. In the first experiment, call-emission rates were quantified under various experimental conditions to establish whether calling becomes more frequent in the dark compared to the light, to determine if the number of calls increases in a more complex environment, i.e. in the presence of obstacles, and to investigate if rearranging obstacles the fish have possibly become familiar with also leads to an increase in calling rate. It is known from many weakly electric fishes of the pulse type that the electric organ discharges these species use to actively probe the environment occur at an irregular and relatively low rate under resting conditions and/or in familiar environments. In the presence of novel stimuli, however, one can observe an increase in both, regularity and pulse-emission rate in these fishes (e.g. [24]–[26]). A comparable phenomenon can also be observed in echolocating bats and dolphins, where animals again increase their call-emission rate as compared to a baseline activity if they approach a target or if the complexity of an environment increases (e.g. [27], [28]). A similar modulation of the call-emission pattern in *A. seemanni*, in our opinion, would be a strong indication that its vocalizations play a role in acoustic obstacle detection.

The first experiment took place in a fish tank, where several environmental factors might have had an effect on the call-emission rate, as will be discussed. To control for these factors, the second experiment took place in a circular, acoustically transparent environment that was lowered into a larger pool. Again, individual fish were confronted with different obstacle conditions. Here, call-emission rates were investigated in dependence on obstacle condition and distance between fish and obstacle.

In the third experiment, individual fish were tested in a Y-maze to check for an alternative function of the calls of *A. seemanni* in intraspecific communication/school formation. Within the maze, the fish had to choose between an arm with an underwater loudspeaker playing back the calls of an artificial school of conspecifics and an arm with a silent loudspeaker dummy. We hypothesized that, if the described calls play a role in school formation, fish should show a preference for the arm with the underwater loudspeaker.

## Materials and Methods

### Ethics Statement

The experiments reported here comply with the NIH-guidelines for the care and use of laboratory animals (6th revision, 1996) and with the current German animal protection law. The keeping and breeding of the experimental animals and their use in non-invasive behavioral studies was approved by the Landeshauptstadt Hannover, Fachbereich Recht und Ordnung, Gewerbe und Veterinärangelegenheiten (No. 42500/1H).

### Experimental Animals

The animals of the present study were obtained from an ornamental fish wholesale (Aquarium Glaser, Rodgau, Germany) and thereafter kept in fish tanks of 100×40×40 cm (width×height×depth) at 24.5°C in a light-proofed room of our laboratory. Water salinity was adjusted to be the same as used by Aquarium Glaser by adding 3.125 g of sea salt per liter of tap water, corresponding to mildly brackish water. Further, fish were exposed to an artificial light/dark (LD) cycle of 12∶12 h by using a digital clock timer and a set of standard neon tubes. Total body lengths (TL, for definition see [29]) of our specimens of *Ariopsis seemanni* ([17], [30]) were around 12 cm, corresponding to approximately one third of the adult size [31].

### Recording Environments

For the sound recordings, we used two different environments, a fish tank that was identical to the holding tanks and a substantially larger, circular pool with a diameter of 3.66 m and a depth of 0.7 m. The pool was set up in an automatically temperature-controlled room on a heat-insulating polystyrene floor. The pool-water was passively heated to a constant temperature of 24.5°C through the surrounding air.

### Sound Recordings

All fish vocalizations were transduced via a hydrophone (Type 8103, Brüel & Kjær), amplified (Conditioning Amplifier 2626, Brüel & Kjær) and band-limited (70–3,000 Hz; Dual Variable Filter VBF 42 M, Kemo) prior to digitization. For the generation of WAV (Waveform Audio File Format) files, pre-processed signals were fed into a sound card (DS-XG Audio (WDM), Yamaha; amplitude resolution: 16 bit) operated at a sampling rate of 11,025 Hz and subsequently stored on a personal computer (PC). In parallel, for real-time monitoring, analogue signals were applied to a second PC (hardware: DSP Board PC56D, Ariel; software: Sona-PC, Waldmann), an audio monitor, and an oscilloscope (HM 205-2, Hameg). Together, the resulting sonagrams (displays of frequency vs. time vs. relative amplitude) and the output of the audio monitor reliably enabled the experimenter to audio-visually determine the number of calls emitted.

### Sound Characterization

For their physical characterization, we recorded fish calls under two different behavioral and two different environmental conditions: The first set of calls (n = 50) was recorded from a school of 12 *A. seemanni* that were freely exploring the above-mentioned tank with the hydrophone being located at the center of the rectangular cuboid water volume. Since, with this method, it could not be guaranteed that all of the fish contributed equally to the sample, we additionally recorded and analyzed the calls of seven solitary individuals under the same environmental condition (fish tank) and in the pool (n = 20 calls per fish and environment). To ensure that, within the pool, the fish had the same maximal distance to the hydrophone as during the tank-recordings (approximately 0.5 m), the fish were kept in a sub-compartment of the pool while their vocalizations were recorded. This sub-compartment had a square volume of 8 l and was floating in the exact center of the pool. The edges of the cube were made of thin stainless steel wire, while the “walls” of the compartment consisted of plastic fly screen. The fly screen was considered as being highly acoustically transparent and its effects on the quality of the sound recordings, therefore, as being negligible. In the pool, the hydrophone was located at a distance of 0.15 m from one of the sidewalls of the sub-compartment and at a depth of 0.35 m beneath the water surface.

Based on the above-mentioned WAV files, a total of 330 calls (school/fish tank: n = 50 calls; solitary individuals/fish tank: n = 140 calls; solitary individuals/pool: n = 140 calls) were analyzed in detail according to their total duration and spectral composition (peak and center frequency of the fundamental and the first three harmonics; relative intensity of each harmonic with the peak intensity of the fundamental having been used as 0 dB reference) using SIGNAL™ (Version 4.0, Engineering Design). In addition, we analyzed the calls of a single, solitary fish in dependence on water temperature. For this “varying-temperature” curve, the fish was put into the recording tank at a water temperature of 26°C to which it previously had slowly been adapted. Subsequently, the water temperature was uniformly lowered to 20°C over a time course of twelve hours. During these twelve hours, the water temperature was constantly monitored using a digital thermometer (Precision Pocket Thermometer GTH 175/PT, Conrad Electronic GmbH, Germany; resolution: 0.1°C). Each time the water temperature dropped by 0.1°C, a new WAV file was generated, so that the calls that were emitted during the individual recording periods could later be allocated to the respective temperatures. To control for other factors that might have influenced the frequency of the emitted calls, 36 hours later, we recorded an additional set of calls for a second period of twelve hours, using the same fish but keeping the temperature constant at the holding temperature of 24.5°C. Here, the resulting sound file was split into fifteen-minute intervals, leaving 48 sample points for the “constant-temperature” curve. In total, we analyzed 120 calls under varying temperature conditions and 126 calls under the condition of a constant temperature.

### Behavioral Experiment 1

To investigate the functional significance of the calls described in this study, additional sound recordings were obtained from individual fish (n = 8) under controlled experimental conditions, either during the last 10 minutes of the light phase (i.e. between 9∶50 and 10∶00 am) or during different 10-minute intervals within the dark phase (details below). During data acquisition, the behavior of the fish was continuously monitored via an infrared(IR)-sensitive video system (digital video recorder: 6240E 16ch, AverMedia; camera: B/W CCD Camera Module 1043XA high res, RF Concepts; software: Ipcam Version 5.0.0.0017, www.avermedia.com). IR illumination was provided by two custom-made projectors (filter: IR 1013, Göttinger Farbfilter), which were symmetrically installed above the water surface at a distance of 40 cm. As before, the hydrophone was located at the center of the water volume.

**Figure 1:**
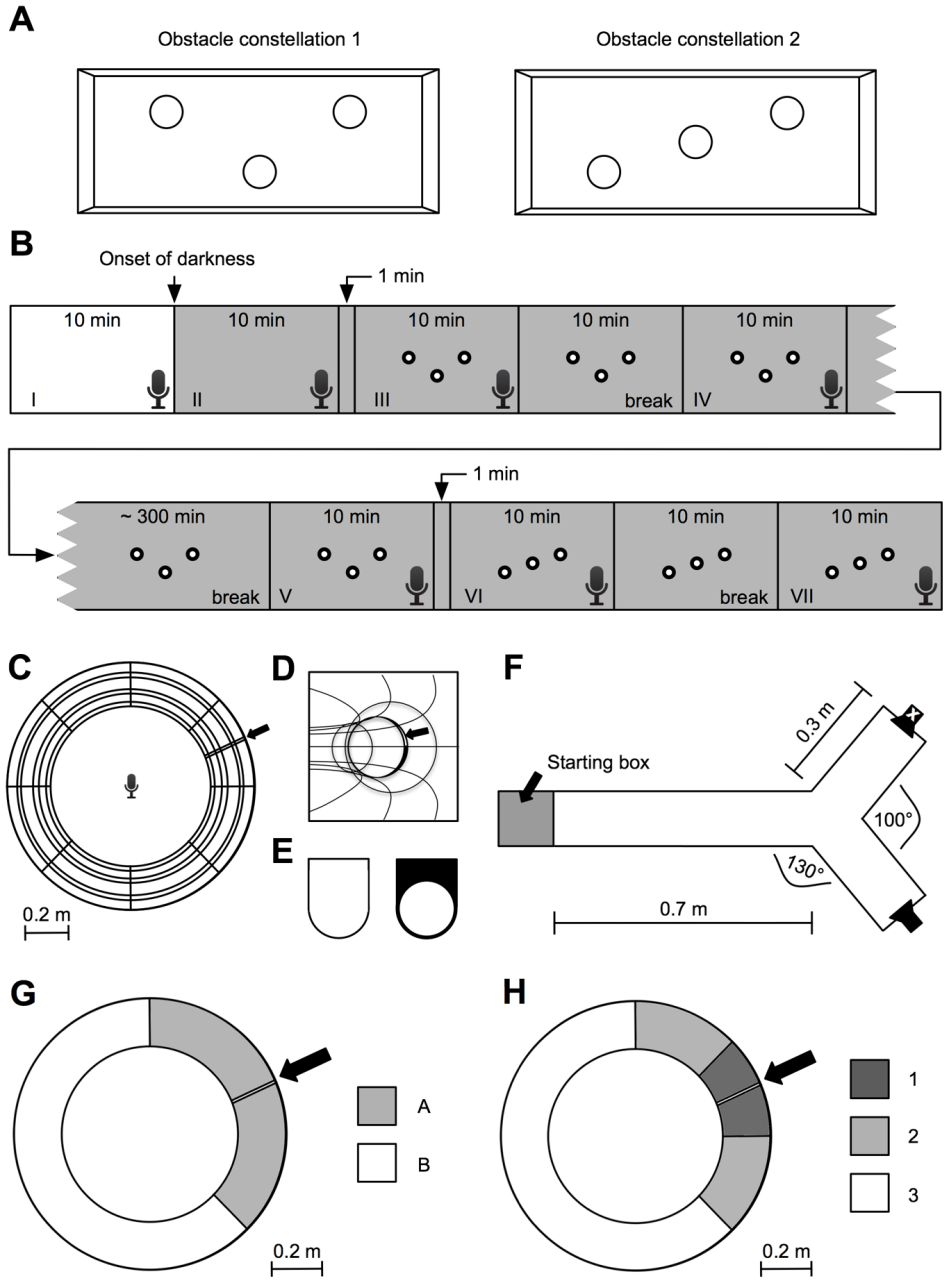
Experimental design. **A** Schematic drawing of the two different obstacle constellations that were used for the behavioral experiment in the aquarium (experiment 1). **B** Timetable for the individual experimental sessions of experiment 1 (I–VII). The small microphone symbols indicate in which of the depicted 10-minute blocks call recording took place. The three small circles within the boxes (III–VII) represent the respective obstacle constellation. Only the first 10 minutes of data acquisition took place under light conditions (white background; I). All other measurements (II–VII) were made in the dark, i.e. under infra-red conditions (gray background). **C** Schematic drawing of the torus-maze used in experiment 2 from a top view (inner diameter = 0.8 m, outer diameter = 1.2 m, diameter of a cross-section of the torus = 0.2 m). The microphone symbol marks the position of the hydrophone, the black arrow points towards the bracket that held the respective obstacle. **D** Schematic drawing of the torus-maze from the inside of the torus volume. The black arrow again points towards the bracket/obstacle position. **E** Two different obstacle-shapes have been used in the experiment, one that completely blocked the maze-volume (left, “glass” and “acrylic glass”) and one that narrowed the cross-section of the maze by 2 cm (right, “black ring”). **F** Y-maze used in experiment 3. The gray area indicates the staring box in which the experimental animals had to wait prior to each trial. The base of the maze had a length of 0.7 m, the distance between the two sidewalls was 0.15 m. The two branches, between which the animals had to decide, left the base at angles of 130° and had a length of 0.3 m each. The loudspeaker symbols indicate the positions of the loudspeaker from which the artificial school recording was played back (normal speaker symbol) and the loudspeaker dummy (crossed out speaker symbol). The positions of loudspeaker and loudspeaker dummy varied pseudo-randomly across trials (for details, see “Materials and Methods”). **G** Proximity zones (A and B) that were used for the first off-line video analysis of the data from experiment 2 with zone A symmetrically surrounding the obstacle (marked by the black arrow) and zone A and zone B being of comparable size. **H** Proximity zones (1–3) that were used for the second, more detailed analysis with zone 1 symmetrically surrounding the obstacle (marked by the black arrow) and zone 1 and zone 2 being of substantially smaller size than the most distant zone 3.

Within the recording tank, the individual fish (n = 8), especially their call-emission rates, were studied under the following conditions (10-minute intervals: **I–VII**, Fig. 1B): **I**, light on; **II**, light off; **III**, light off with obstacle constellation 1 (Fig. 1A); **IV**, light off with obstacle constellation 1 (starting 10 minutes after termination of the prior condition); **V**, light off with obstacle constellation 1 (5 hours after prior condition); **VI**, light off with re-arranged obstacles (i.e. obstacle constellation 2; Fig. 1A); **VII**, light off with obstacle constellation 2 (starting 10 minutes after termination of the prior condition). The obstacles (compare Fig. 1A) consisted of 3 transparent and water-filled glass cylinders of identical dimensions (height: 33 cm, diameter: 9 cm).

### Behavioral Experiment 2

The second experiment was conducted in a torus-shaped “maze” (Fig. 1C). Just like the sub-compartment of the pool that was used for the sound recordings (*vide supra*), the maze mainly consisted of plastic fly screen being supported by stainless steel wires. The circular cross-section of the maze-volume at any given place had a diameter of 0.2 m, so that the fish (n = 7) had enough space to swim through the maze without touching the “walls” as well as to turn and change direction if intended. The inner diameter of the torus itself was 0.8 m, resulting in a minimum distance of 2.51 m that had to be swum for one round. At one position along the torus, a bracket allowed for the introduction of obstacles into the volume of the maze (Fig. 1C, D). As a whole, the maze was sunk into the aforementioned pool and was floating approx. 0.2 m beneath the water surface. For visual and acoustic monitoring of the experiments, we used the same equipment as described before with the hydrophone being located at the center of the torus at a depth of 0.35 m.

Once an experimental animal was put into the maze, it was given 30 minutes to habituate to the new environment. After this time, we started to consecutively introduce different kinds of obstacles into the maze-volume: (i) a semi-circular pane made of translucent glass, (ii) a semi-circular pane made of translucent acrylic glass, and (iii) a ring made of black plastic (Fig. 1E). The first two obstacles completely blocked the cross-section of the maze at the site of the bracket, while the third obstacle allowed for a free passage (the maze diameter narrowed to 0.18 m at the site of the plastic ring) and mainly provided visual stimulation. Each obstacle was presented for the duration of 15 minutes and three times per fish. Obstacle installation occurred in a pseudo-randomized fashion and with a pause of 15 minutes between successive trials. After the last obstacle presentation in a given session, the illumination was turned off and the call-emission rate was monitored in complete darkness for a final interval of 15 minutes.

### Behavioral Experiment 3

The Y-maze for the third behavioral experiment also was set up within the pool. The walls of the maze were made of white plastic boards (distance between the walls = 0.15 m), with the two branches of the maze leaving the base at an angle of 130° (Fig. 1F). The base had a length of 0.7 m, while the branches had a length of 0.3 m each. At the end of one of the branches (changing pseudo-randomly during the experimental sessions, see below), an underwater loudspeaker (Model MA001, DARAVOC) was installed from which the calls of an artificial school of conspecifics were played back into the water at a natural sound-pressure level. For the generation of this “artificial-school” sound, we used a real school recording as a template and constructed a WAV file containing 100 “high-quality” calls from our call database and imitating the temporal pattern of the template. This sound-file was looped to a length of approx. 1 hour and fed into the loudspeaker via an audio amplifier (Integrated Amplifier SU-V300, Technics). To electro-magnetically shield the loudspeaker, it was wrapped in a thin, grounded silver-wire mesh that served the function of a Faraday cage. At the end of the opposite branch, we installed a loudspeaker dummy that was a copy of the real loudspeaker in both color and form and also wrapped in a grounded silver-wire mesh.

At the beginning of each trial, the individual experimental animal (n = 5) was put into a starting box at the front end of the base of the maze (Fig. 1F). Once the fish had habituated to the box, the wall leading to the base of the Y-maze was removed and the fish was allowed to freely explore the maze. After it made a decision for one of the branches of the maze (by swimming into the respective branch), the experimental animal was re-captured and put into an opaque fish tank until the next trial was prepared. In total, each fish completed 20 of these trials. The position of the loudspeaker was changed pseudo-randomly between trials (10 left, 10 right; the loudspeaker never occurred for more than three successive trials at the same position).

### Data Analysis

As mentioned above, the sound analysis for the physical characterization of the calls was done using SIGNAL™ (Version 4.0, Engineering Design). For the analysis of the sound recordings from the first two behavioral experiments, we used the open-source audio editor Audacity (Version 2.0.2) to visually (via sonagrams) and acoustically identify calls and to determine the exact time points of their emission. The video data from behavioral experiment 2 were processed using ImageJ (Version 1.46, Wayne Rasband, National Institute of Health) for a frame-to-frame analysis. In order to investigate the call-emission rates of the experimental animals in dependence on the distance between the individual fish and the installed obstacles, the torus-maze was virtually divided into proximity zones. For a coarse analysis, two zones were defined (A and B; Fig. 1G), a more detailed analysis included three proximity zones (1–3; Fig. 1H).

The acquired data were analyzed using the free programming environment R (R 2.11.1, 2010, The R Foundation for Statistical Computing) for descriptive statistics as well as for hypothesis testing and plotting of the data. To test the different temporal and spectral sound characteristics (call duration, number of harmonics, peak and center frequencies) for normality, a battery of the most common tests for normality (Shapiro-Francia, Anderson-Darling, Cramer-von Mises, Kolmogorov-Smirnov, and Pearson chi-square normality test) was applied. Since the Null-Hypothesis of normal distribution had to be rejected in many cases, we exclusively used non-parametric statistics for data description (median, upper and lower hinge, interquartile range) and for hypothesis testing.

## Results

### Call Parameters

**Figure 2:**
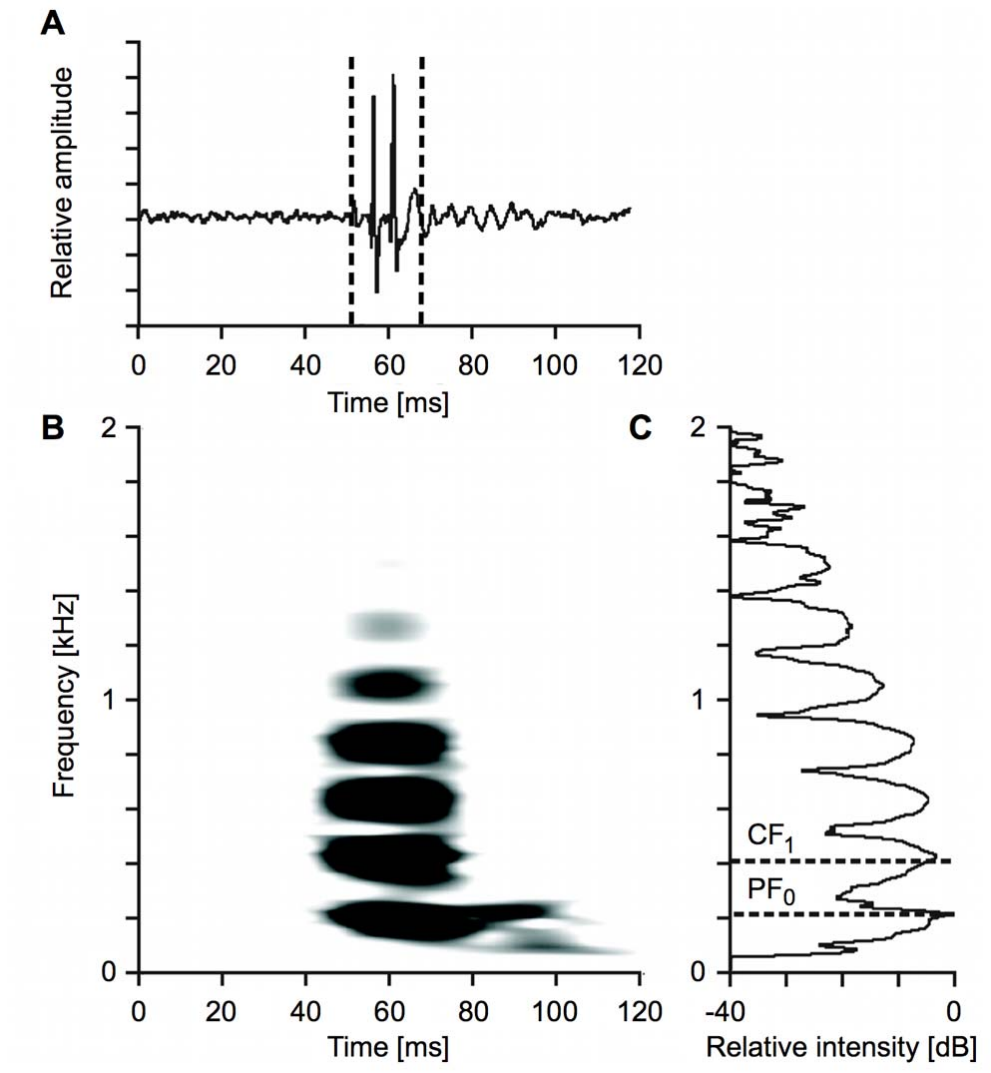
Exemplary depiction of a constant-frequency call of *A.* seemanni. **A** Oscillogram of the signal. The length of the segment between the dashed lines corresponds to the call duration as it was measured from the oscillogram in combination with the information from the respective sonagram, which had its emphasis on the temporal resolution (not shown here). **B** Sonagram of the same signal with enhanced frequency resolution (set to 21.5 Hz) illustrating the multi-harmonic and tonal structure of the call. **C** Power spectrum of the signal. The dashed lines exemplify how the spectral measurements were performed. The determination of the peak frequency (PF) is shown for the fundamental (PF_0_), the one of the center frequency (CF) for the first harmonic (CF_1_). Other measurements (PF_1_–PF_7_; CF_0_ and CF_2_–CF_7_) were obtained accordingly (compare “Materials and Methods”).

**Figure 3:**
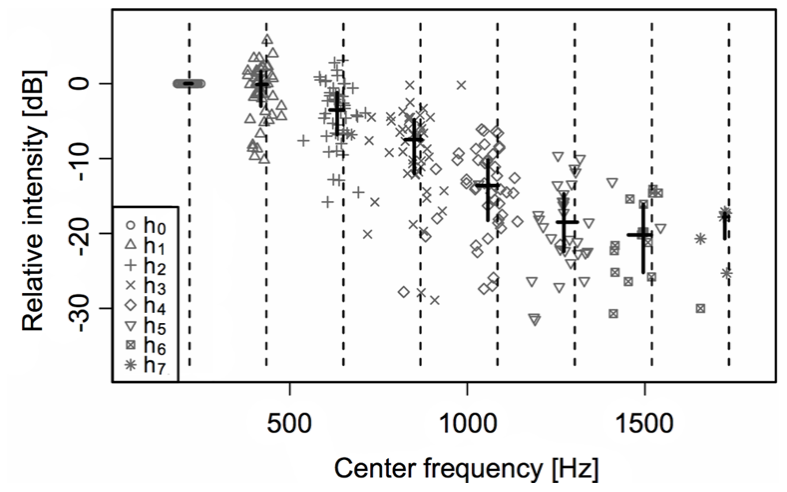
Center frequencies and relative amplitudes (school, aquarium). Distributions and central tendencies of the center frequencies and relative amplitudes (using the amplitude of the belonging fundamental frequencies as a 0 dB reference) of the fundamental (h_0_) and the different harmonics (h_1_–h_7_). Dashed lines correspond to integer multiples of the median center frequency of the fundamental (217.05 Hz). Horizontal solid lines indicate the interquartile range of the center frequencies of the fundamental and the respective harmonics, vertical solid lines the belonging interquartile range of the relative amplitudes. For each harmonic and the fundamental, the pair of solid lines crosses at the respective medians of the represented parameters. From the fundamental frequency to the sixth harmonic, there is a strong linear relationship between the order of the harmonic (0–6) and the respective relative amplitude (adjusted R^2^ = 0.9526, for reasons of clarity, the regression line is omitted).

Independent of the recording condition (pool vs. aquarium, school vs. solitary fish), the call type of *A. seemanni* analyzed in this study can be described as a short, multi-harmonic constant-frequency call (Fig. 2B) with a median duration of 19.5 ms (compare Fig. 2A for the oscillogram of an exemplary call). The main energy of the calls lies on the fundamental frequency (h_o_) and the first harmonic (h_1_). Between the harmonics 0 and 6, peak energy linearly decreases towards the higher harmonics (Figs. 2C and 3). The minimal fundamental frequency measured in our specimen of *A. seemanni* at 24.5°C was 148.3 Hz, the maximal fundamental frequency measured under the same temperature conditions was 249.8 Hz.

Within the school of 12 individuals, from which the randomly sampled calls (n = 50) have been recorded in an aquarium, the peak frequencies of the calls’ fundamentals range from 205.5 Hz (lower hinge) to 223.8 Hz (upper hinge; median = 217.4 Hz). Comparably, the center frequencies of the fundamentals range from 204.4 Hz (lower hinge, f_min_ = 178.2 Hz) to 224.7 Hz (upper hinge; median = 217.05 Hz, f_max_ = 249.8 Hz; Fig. 3). The median number of detectable harmonics is 6. The analyzed signals show a strong linear relationship between the order of the harmonic (0–6) and the respective median relative amplitude in dB (adjusted R^2^ = 0.9526; Fig. 3). The slope of the resulting regression line (not shown) corresponds to −10 dB/565.34 Hz.

The seven fish from which sounds were recorded in isolation and under two different environmental conditions (fish tank and pool) show median center frequencies of the fundamental ranging (lower to upper hinge) from 180.425 Hz to 199.4 Hz (f_min_ = 155.35 Hz, f_max_ = 222.5 Hz) in the aquarium and from 177.55 Hz to 206.1 Hz (f_min_ = 148.3 Hz, _max_ = 219.55 Hz) in the pool (compare Table 1 and Figs. S1, S2, S3, S4, S5, S6, S7). Significant differences in the spectral composition (center frequencies) of the calls between the pool recordings and the aquarium recordings were found for fish 2 (1^st^ harmonic: p = 0.0093; 2^nd^ harmonic: p = 0.00027; 3^rd^ harmonic: p = 0.0002; Wilcoxon rank sum test, two-sided, confidence level = 0.95, p-values “Holm-Bonferroni”-adjusted for multiple testing) and fish 3 (1^st^ harmonic: p = 0.0039; Wilcoxon rank sum test, details as before). Concerning the center frequencies of the fundamentals, no significant differences were found between the two recording environments for any fish (Figs. S1, S2, S3, S4, S5, S6, S7).

**Table 1:** Comparison of the individual median center frequencies of the fundamental (0) ant the first three harmonics (1–3) between the recording environments (aquarium *vs.* pool).

Subject	Median center frequency [Hz] (Aquarium)	Median center frequency [Hz] (Pool)	Harmonic
Fish 1	187.475	180.725	0
	351.85	346.8	1
	518.6	510.525	2
	703.075	683.85	3
Fish 2	186.2	191.375	0
	350.9	** 362.45	1
	524.575	*** 555.7	2
	708.475	*** 739.325	3
Fish 3	186.5	189.7	0
	380.4	** 359.875	1
	566.95	562.775	2
	737.4	731.6	3
Fish 4	199.4	206.1	0
	387.25	383.625	1
	591.8	598.725	2
	796	796.425	3
Fish 5	183.65	180.1	0
	350.875	355.325	1
	528.425	539.55	2
	700.475	726.075	3
Fish 6	187.825	184.9	0
	382.65	375.275	1
	564.7	574.95	2
	747.675	770.625	3
Fish 7	180.425	177.55	0
	342.2	347.85	1
	514.4	523.9	2
	695.675	695.975	3

Significance code:

** p<0.01;

*** p<0.001.

**Figure 4:**
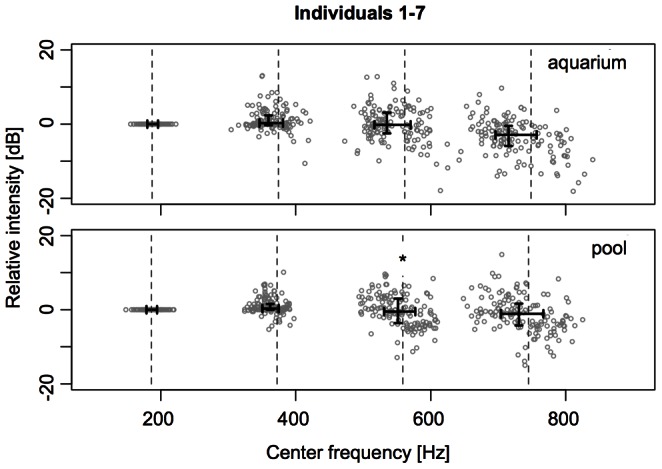
Center frequencies and relative amplitudes (aquarium *vs.* pool). Distributions and central tendencies of the center frequencies and relative amplitudes (using the amplitude of the belonging fundamental frequencies as a 0 dB reference) of the fundamental and the first three harmonics. Dashed lines correspond to integer multiples of the median center frequency of the fundamental. Horizontal solid lines indicate the interquartile range of the center frequencies of the fundamental (h_0_) and the first three harmonics (h_1_–h_3_), vertical solid lines the belonging interquartile range of the relative amplitudes. For the fundamental and each harmonic, the pair of solid lines crosses at the respective medians of the represented parameters (significance code: *p<0.05; Wilcoxon rank sum test, two-sided, confidence level = 0.95, p-values “Holm-Bonferroni”-adjusted for multiple testing). Group-level comparison (pooled data from individual 1–7; compare Figs. S1, S2, S3, S4, S5, S6, S7 for individual comparisons) of the measured parameters between the two recording environments (aquarium and pool).

When the data of all fish are pooled, as in an hypothetical sound recording from a school to which all individuals contribute the same number of calls, the center frequencies of the fundamentals range from 180.1 Hz (lower hinge) to 195.975 Hz (upper hinge; median = 187.15 Hz) in the aquarium and from 178.8 Hz (lower hinge) to 194.55 Hz (upper hinge; median = 186.2 Hz; Fig. 4) in the pool. Here, significant differences in the spectral composition (center frequencies) of the calls between the recording environments could only be found for the 2^nd^ harmonic (p = 0.04908; Wilcoxon rank sum test, two-sided, confidence level = 0.95, p-values “Holm-Bonferroni”-adjusted for multiple testing).

**Figure 5:**
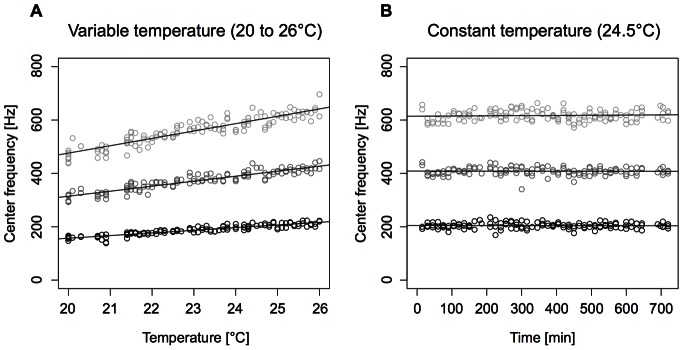
Temperature dependence of the calls recorded from a solitary fish. **A** Center frequencies of the fundamental (black) and the first two harmonics (dark gray: 1^st^ harmonic, light gray: 2^nd^ harmonic) measured under varying temperature conditions (steady decrease of the temperature from 26°C to 20°C over a period of 12 hours). The center frequencies of the fundamental and the harmonics correlate highly significantly with water temperature (r-values ranging from 0.91 to 0.92; p-values <2.2*10^−16^). **B** Center frequencies of the fundamental (black) and the first two harmonics (dark gray: 1^st^ harmonic, light gray: 2^nd^ harmonic) measured under constant temperature conditions (at 24.5°C) for control purposes. When the temperature is kept constant, frequencies fluctuate around a stable median.

Within the additional call data from a solitary fish, recorded in the aquarium for 12 hours under varying water-temperature conditions and for twelve hours under the condition of a constant water temperature, the center frequencies of the fundamental as well as the harmonics highly significantly correlate (fundamental: r = 0.913, p<2.2*10^−16^; 1^st^ harmonic: r = 0.922, p<2.2*10^−16^; 2^nd^ harmonic: r = 0.913, p<2.2*10^−16^) with the value of the water temperature at the time point of call emission (Fig. 5A). In our example (within the range between 26 and 20°C), lowering the water temperature by 1°C caused a decrease in the calls’ fundamental of approx. 10 Hz. At 20°C, the center frequencies of the fundamental vary around 156.4 Hz (mean), while at 26°C they vary around 220.9 Hz (mean). When the temperature is kept constant, however, the center frequencies of the fundamental and the harmonics of the emitted calls also stay constant over time (fundamental: r = −0.019, p = 0.83; 1^st^ harmonic: r = −0.019, p = 0.83; 2^nd^ harmonic: r = 0.071, p = 0.43). Here, at a constant temperature of 24.5°C, the center frequencies of the fundamental fluctuate around 204.2 Hz (mean; Fig. 5B). No significant correlation was found between the center frequencies of the individual fundamentals from the pool recordings and the body lengths of the fish (r = −0.122, p = 0.79).

### Behavioral Experiment 1

**Figure 6:**
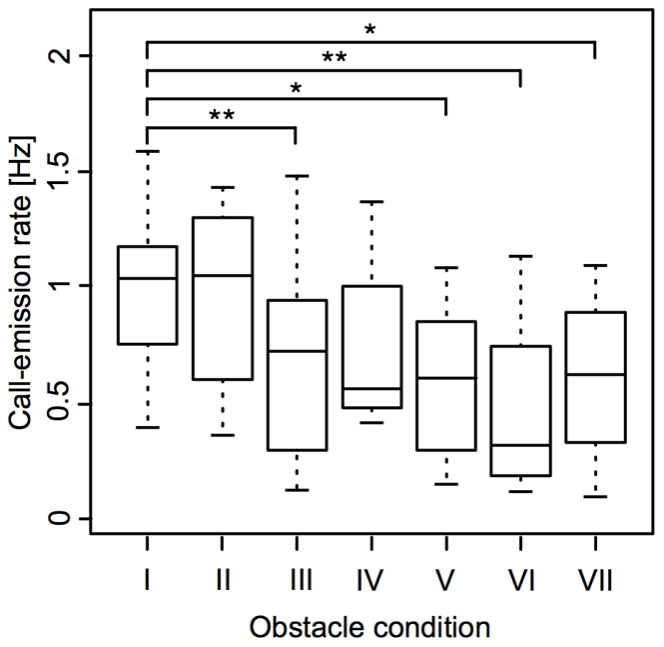
Call-emission rates under the different experimental conditions of experiment 1 (I – VII, compare Fig. **1B).** There are no significant changes in the call-emission rates between the obstacle-free conditions in the light (I) and in the dark (II). However, the call-emission rate decreases as soon as obstacles are introduced into the aquarium (III–VII). This decrease in the call-emission rate as compared to the obstacle-free light condition (I) was significant in most cases (III, V – VII, Wilcoxon signed rank test for paired data, two-sided, confidence level = 0.95) as indicated by the asterisks (significance code: *p<0.05; **p<0.01).

Under all of the experimental conditions (dark, obstacle constellation I, obstacle constellation II; compare Fig. 1A, B), no significant (Wilcoxon signed rank test for paired data, one-sided, confidence level = 0.95) increase in the call-emission rate as compared to that under the light condition could be observed (Fig. 6). When only the light was switched off (see “onset of darkness” in Fig. 1B), the median call-emission rate stayed virtually the same and no significant difference between the two conditions (light vs. dark) could be found (Wilcoxon signed rank test for paired data, two-sided, confidence level = 0.95, p = 0.53; Fig. 6). Under those conditions where obstacles were introduced into the aquarium (**III** – **VII**; Figs. 1B and 6), the call-emission rate, in contrast to our hypotheses (see “Introduction”), significantly *decreased* as compared to that under the obstacle-free light condition (Wilcoxon signed rank tests for paired data, two-sided, confidence level = 0.95, p-values ranging from 0.0078 to 0.0234; exception: **I** vs. **IV**: p = 0.0547; Fig. 6).

### Behavioral Experiment 2

**Figure 7:**
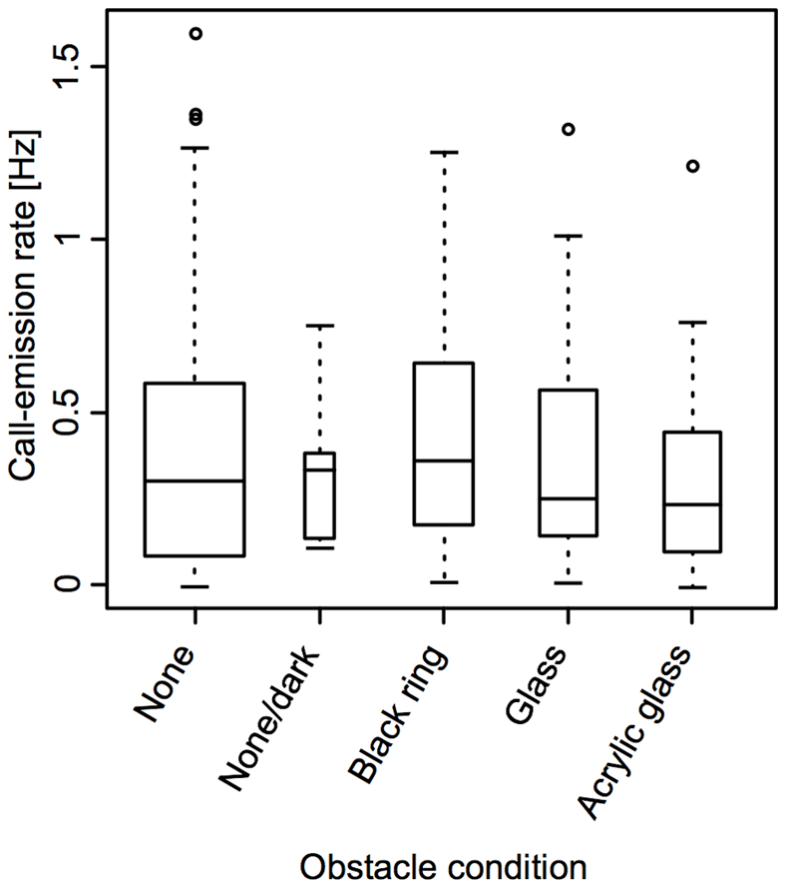
Call-emission rates under the different obstacle conditions of experiment 2. There are no significant changes in the call-emission rates between the obstacle-free condition in the light and the other obstacle conditions (p-values ranging from 0.40 to 0.97, Wilcoxon rank sum test, two-sided, confidence level = 0.95).

Based on the video- and sound-documentation of experiment 2, one could see that almost every time the experimenter approached the maze, the respective experimental animal immediately stopped its motor activity as well as call emission until the experimenter left. The calculations of the call-emission rates for the different obstacle conditions, therefore, do not include the time intervals 30 s before and after an obstacle was installed or removed. The so-determined call-emission rates under the different obstacle conditions and in the dark (Fig. 7) do not significantly differ from the call-emission rates under the obstacle-free condition in the light (no obstacle vs. dark: p = 0.97; no obstacle vs. black ring: p = 0.76; no obstacle vs. glass obstacle: p = 0.97; no obstacle vs. acrylic glass obstacle: p = 0.4; Wilcoxon rank sum test, two-sided, confidence level = 0.95).

**Figure 8:**
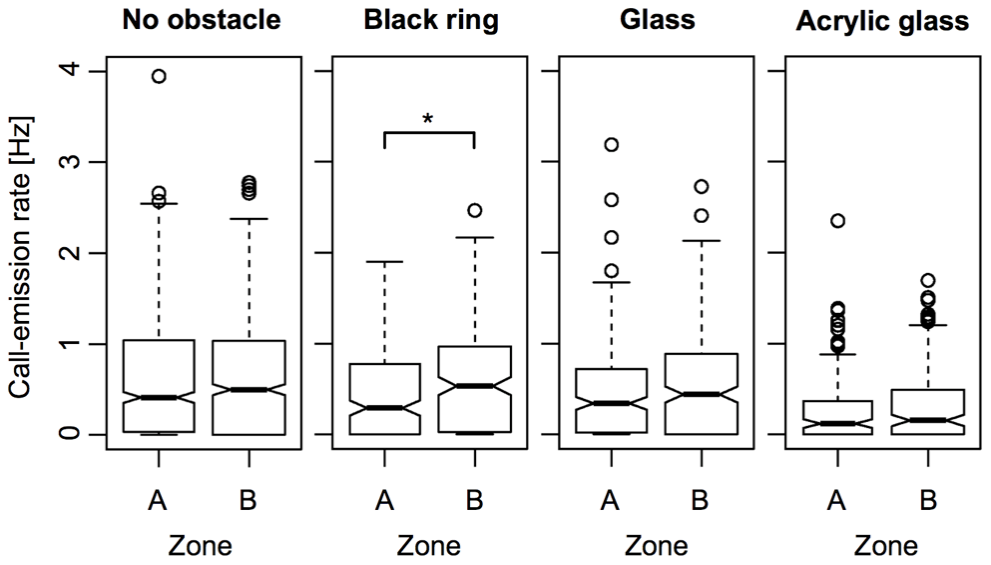
Call-emission rates under the different obstacle conditions of experiment 2 and in dependence on the proximity of the experimental animals to the respective obstacle (analysis 1: zones A and B; compare Fig. 1G). The width of each boxplot is proportional to the square root of the number of observations in the respective zone. The notches extend to +/−1.58 IQR/sqrt(n) and approximate a 95% confidence interval around the belonging median (bold horizontal line). Significant differences in the call-emission rates between the proximity zones have only been found for the “black-ring” obstacle (p = 0.011, Wilcoxon rank sum test, two-sided, confidence level = 0.95; significance code: *p<0.05).

When comparing the call-emission rates for each obstacle condition in dependence on the proximity of the fish to the obstacle, significant differences between the call-emission rates in proximity zones A and B (Fig. 1G) can only be found for the “black ring” condition (p = 0.011, Wilcoxon rank sum test, two-sided, confidence level = 0.95; Fig. 8). The belonging medians (A: 0.29 Hz, B: 0.53 Hz) of the call-emission rates differ by 0.24 Hz (Fig. 8). For the remaining obstacle conditions, no significant differences in the call-emission rates exist between the proximity zones A and B (p-values range between 0.37 and 0.85, Wilcoxon rank sum test, two-sided, confidence level = 0.95, Fig. 8).

**Figure 9:**
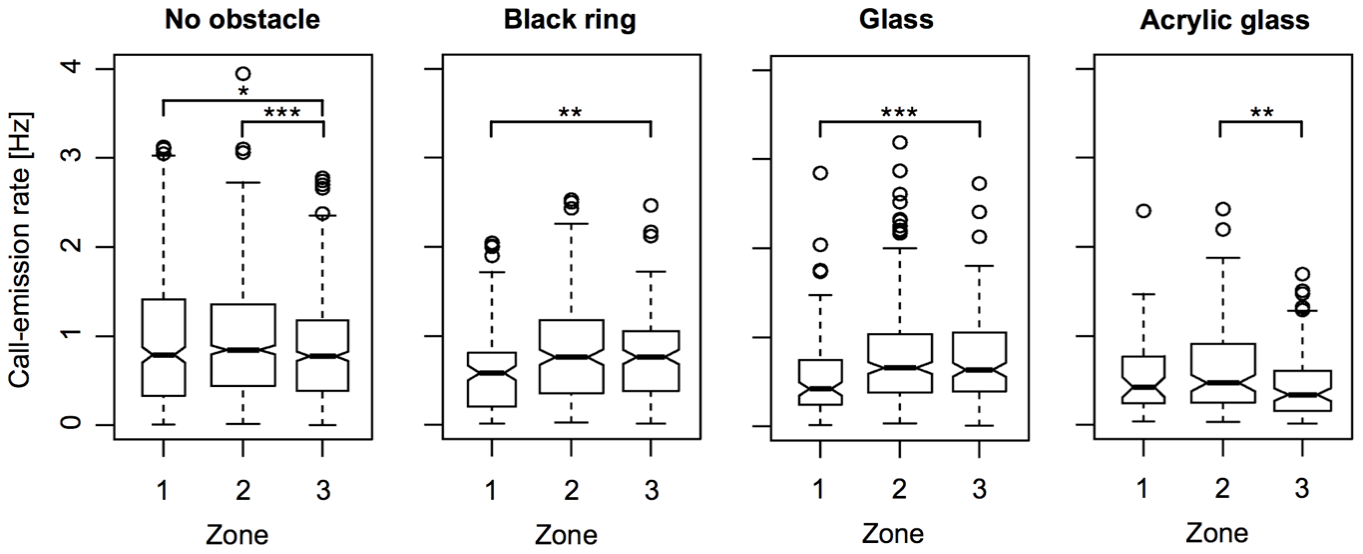
Call-emission rates under the different obstacle conditions of experiment 2 and in dependence on the proximity of the experimental animals to the respective obstacle (analysis 2: zones 1 to 3; compare Fig. 1H). The width of each boxplot is proportional to the square root of the number of observations in the respective zone. The notches extend to +/−1.58 IQR/sqrt(n) and approximate a 95% confidence interval around the belonging median (bold horizontal line). Significant differences in the call-emission rates between proximity zone 3 and 2 have been found for the “no-obstacle” and the “acrylic-glass-obstacle” conditions. Between zone 3 and 1, significant differences in the call-emission rates have been found for the obstacle conditions “glass”, “black ring”, and “no obstacle” (significance code: *p<0.05; **p<0.01; ***p<0.001; Wilcoxon rank sum test, two-sided, confidence level = 0.95).

The more detailed analysis of the call-emission rates for each obstacle condition using a different set of proximity zones (1, 2, and 3; Fig. 1H) revealed the problem that, due to the small length of the two most proximate zones (1 and 2), the experimental animals stayed for significantly shorter time intervals in these zones than in proximity zone 3. Consequently, there was a much higher chance in zone 1 and 2 for the occurrence of call-emission rates of 0 Hz (when no call was uttered while being in the respective zone). We, therefore, omitted all values of 0 Hz to correct for this bias. In the remaining data, significant differences (Wilcoxon rank sum test, two-sided, confidence level = 0.95) exist between the call-emission rates in proximity zones 2 and 3 for the obstacle conditions “no obstacle” (p = 0.00028, lower call-emission rate in 3, Δη = 0.0719 Hz) and “acrylic glass obstacle” (p = 0.0061, lower call-emission rate in 3, Δη = 0.0844 Hz) and between the call-emission rates in proximity zones 1 and 3 for the obstacle conditions “no obstacle” (p = 0.024, lower call-emission rate in 3, Δη = 0.0163 Hz), “glass obstacle” (p = 0.0003, higher call-emission rate in 3, Δη = 0.2145 Hz), and “black ring” (p = 0.0041, higher call-emission rate in 3, Δη = 0.1806 Hz; Fig. 9).

### Behavioral Experiment 3

**Figure 10:**
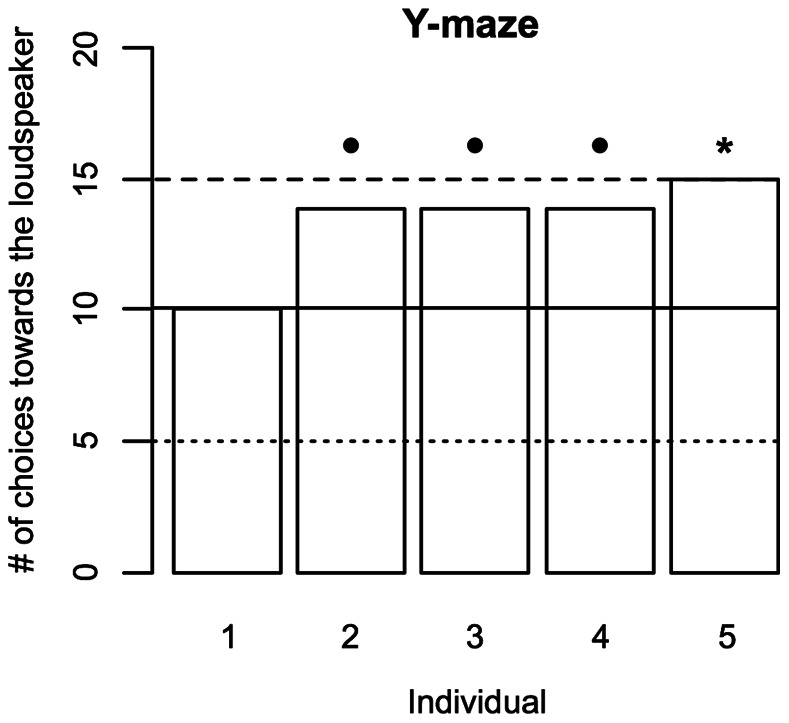
Results of behavioral experiment 3. The bar chart depicts the number of decisions each experimental animal made for the arm of the Y-maze containing the loudspeaker (dotted line: significant number of decisions against the loudspeaker; solid line: chance level; dashed line: significant number of decisions for the loudspeaker; significance code: •p<0.1; *p<0.05).

Of the 5 fish that participated in the Y-maze experiment, one individual performed exactly at chance level (50% of choices made for the arm with the loudspeaker). Three other fish almost reached the significance criterion for a deviation from chance level by choosing the arm with the loudspeaker in 70% of the trials (n = 20, cumulative binomial probability: p = 0.058), while the last one did reach criterion (75% of the trials, n = 20, cumulative binomial probability: p = 0.021; Fig. 10). In total, the fish decided in 67% of the trials for the arm with the loudspeaker (n = 100, cumulative binomial probability: p = 0.00044).

## Discussion

### Recording Quality

The detailed analysis of the short, constant-frequency calls of *A. seemanni* in this study is based on sound recordings from both a glass aquarium and a substantially larger pool. Whether spatially restricted environments like aquaria allow for a proper physical characterization of underwater animal vocalizations, is controversially discussed in the literature (e.g. [32]). However, recent studies (e.g. [33], [34]) clearly point towards the suitability of such aquarium recordings. For example, in the catfish *Orinocodoras eigenmanni* it was found that even minor waveform details persist when sounds derived from a fish tank were compared to those recorded in the field from the same individuals [33].

The results presented here speak in favor of this position. In a comparison of the calls from a group of 7 individuals between the two environmental conditions (aquarium *vs.* pool), no significant differences in the spectro-temporal composition of the emitted calls were found except for the second harmonic (caused by a positive skew in the distribution of the aquarium sample). On the individual level, however, such differences existed in two of seven animals, but they only concerned some of the harmonics and never the fundamental, which usually is the frequency of most importance for the spectral appearance (i.e. spacing of the harmonics; e.g. [35]) of tonal, multi-harmonic sounds. We, therefore, conclude that, for the study at hand, the size of the recording environment did not play a noteworthy role for physical call characterization and we are confident of having properly described the spectro-temporal nature of the swim-bladder sounds of *A. seemanni*. However, we would like to remark that when recording the sounds for a call characterization in an aquarium, great care has to be taken to keep the environmental parameters constant during recording. For example, we could show that the spectral composition of the calls of *A. seemanni* highly depends on the surrounding water temperature, or causatively on the animal’s body temperature, and comparable results have also been found in other vocal, teleost fishes [36]. Normally, to achieve high quality, noise-free recordings in a fish tank, all supporting equipment, such as oxygen-pump and heater, has to be switched off. Thus, longer recording sessions can lead to a non-negligible drop in water-temperature that influences the measured results. Due to the substantially larger water-body, the physical parameters of the pool environment are much more stable, which is why such an environment should be preferred over an aquarium if available.

Regarding the two behavioral conditions, under which the emitted calls have been recorded in the aquarium for call characterization, a direct comparison of the frequency composition of the calls between the conditions “school” vs. “solitary fish” was not possible, since both samples were composed of different individuals (school: n = 12, solitary fish: n = 7) with the calls showing a relatively high inter-individual variability. However, comparing the differences between the maximum and the minimum of the measured fundamental center frequencies between the samples (school: Δf = 71.59 Hz, solitary fish: Δf = 67.15 Hz) indicates that both sampling methods are suited equally well for getting an impression of the variance of the sound characteristics within a population. The danger that, in a sample of randomly drawn calls uttered from a school of fish, single individuals might contribute to unequal amounts seems to be negligible as long as the sample size is large enough.

### Behavioral Experiments 1 and 2

In experiment 1, where illumination conditions and the presence or absence of obstacles have systematically been modified in order to induce changes in the call-emission rates of the individual fish, statistical results are not significant in the direction of the stated hypotheses (e.g. an expected increase in calling rate in the dark or in the presence of obstacles; compare “Introduction”). However, in the presence of obstacles, call-emission rates significantly (confidence level = 0.95) decreased as compared to the light or obstacle-free dark condition. In the more complex environments, fish were often found turning around and around within a small area of the glass aquarium. Tavolga [12] reported the latter behavior upon the addition of a complex arrangement of plastic barriers also for single, intact *A. felis*. The call-emission rate, therefore, might correlate with locomotory activity instead of obstacle condition. Unfortunately, the infrared light conditions did not allow for a gapless off-line analysis of the experimental animals’ behavior from the video recordings made during the experiment to clarify this matter. This however, was possible for experiment 2. As compared to the aquarium from the first experiment, the torus-maze setup had several advantages that allowed us to control for some of the factors that could have influenced the first experiment: (i) Due to the torus shape of the maze, the distance between the experimental animal and the hydrophone only varied within a small range. (ii) Within the torus volume of the maze, experimental animals were provided with a privileged movement direction. The turning behavior mentioned above could only rarely be observed. In addition, the orientation of the fish in reference to the hydrophone was more constant (i.e. it was mainly the flanks of the fish that pointed towards the hydrophone). (iii) Due to the utilization of only one obstacle at a given time, call-emission rates could be analyzed in dependence on the proximity between fish and obstacle. This was not possible in experiment 1, where the experimental animals constantly were in the vicinity of multiple, differently placed objects. (iv) Since most of the second experiment took place under normal illumination, the video recordings from experiment 2 provide a complete overview of the behavior of the experimental animals in the presence of the obstacles. What we first found, based on the analysis of these videos, was that the experimental animals showed a startle reaction in response to the installation/removal of an obstacle and/or the associated sudden appearance of the experimenter, manifesting itself in a reduction of call emission and motor activity. This startle response can also explain the significant drops in call-emission rate found in experiment 1: In order to install the obstacles into the fish tank, the experimenter had to approach the aquarium. The installation of the obstacles itself temporarily caused disturbances of the water body. Both events might have caused startle reactions as described above and consequently have led to the found decreases in the call-emission rates in experiment 1. Turning off the light, in contrast, was done remotely, explaining why the call-emission rate, here, did not drop. In experiment 2, where intervals of 30 s before and after the installation or removal of an obstacle were excluded from the analysis, significant differences in the call-emission rates between the obstacle conditions disappeared. This finding further substantiates our interpretation of the results from the first experiment.

The investigations of the call-emission rate in dependence on the proximity of the individual fish to the respective obstacle led to diverging results. In the analysis where two proximity zones had been defined, the only significant effect could be detected for the “black-ring” obstacle. In zone A, the call-emission rate significantly dropped (Δη = 0.2421 Hz) as compared to zone B, which was more distant from the obstacle. The more detailed analysis using three proximity zones revealed that this decrease of the call-emission rate was a local event, being restricted to the immediate surrounding (proximity zone 1, Δη = 0.1806 Hz) of the obstacle. Since the experimental animals usually passed the ring-like obstacle without exploring it, it must have been the brief visual stimulation by the black-colored obstacle that caused the reaction of the fish.

Of the four other significant differences that were found in the detailed analysis using proximity zones 1 to 3, three indicated only subtle changes (increases) in the median call-emission rates (Δη <0.1 Hz) in proximity zones 1 and 2 as compared to the most distant zone 3. Two of these subtle but significant changes in call-emission rate were found for the “no-obstacle” condition, so that they cannot be related to the proximity to an obstacle. The third case was found under the “acrylic-glass” obstacle condition, but medians differed significantly between proximity zones 3 and 2 and not between proximity zones 3 and 1 as it would have been expected based on our hypothesis. Hence, it is highly unlikely that this difference in the median call-emission rates under the “acrylic-glass” obstacle condition is actually caused by the presence of the obstacle. In principle, one might have to be cautious with regard to the p-values provided by the Wilcoxon rank sum test for the above-mentioned cases. When calculating the 95% confidence intervals for each median (compare Fig. 9), there still is partial overlap between these intervals, indicating that there is no strong evidence for a difference in central tendency between the compared samples. Even though the comparison of confidence intervals is not a formal statistical test, these contrary results might nevertheless be noteworthy.

The remaining significant difference in call-emission rate we found was under the “glass-obstacle” condition between proximity zone 3 and 1. Here, it was again a decrease in the call-emission rate in zone 1 (next to the obstacle) as compared to zone 3 (Δη = 0.2145 Hz). One possible explanation for this finding is that the experimental animals temporarily stopped vocalizing after they collided with the obstacle, but unfortunately, due to the transparency of the obstacle, it was not possible to strictly distinguish situations where an individual bumped into the obstacle from those where it turned right before the obstacle in the video recordings.

### Acoustic Orientation

We are aware of the fact that the first two behavioral experiments, be it in the aquarium or in the pool, have not been suited to investigate any kind of far-field echolocation as it can be found particularly in bats or toothed whales. However, in his original paper about the possible role of call emissions for obstacle detection and avoidance in *A. felis*, Tavolga [12] already states that, due to signal parameters, such as relatively low frequency and amplitude, it is fairly unlikely that reverberations from the far field contain any useful spatial information. Instead, if existent at all, echoacoustic orientation in catfishes most likely relies on the interpretation of acoustic reverberations in the near field. In order to provide a fish with near-field echoacoustic spatial information, its emitted signals have to fulfill at least two requirements: (i) The signal has to be of relatively high amplitude, so that enough acoustic energy gets reflected and returns to the sender. (ii) The direction of the particle motion carrying the interpretable information (details below) has to be parallel to the axis of the reflecting object and the receiving individual. The fish itself, as the receiver, then has to be able to extract this directional information in order to perceive the location of the reflecting object.

Today, it is widely believed that sound-source localization in fishes *via particle-motion reception* is only possible through the otolith organs [37]–[39], where each hair cell has a directional-sensitivity vector for maximal depolarization [40]. When directional hearing alone is considered (i.e. direct transmission from a sound source to the receiver), Popper and colleagues [41] stated that particle-motion processing is most likely at low frequencies (below 300 Hz), at high sound-pressure levels (above 80 dB: re 1 μPa), and only in direct proximity (d <1 λ) to the sound source. As already mentioned above, an important assumption that is made in these considerations is that the axis of particle motion and the directional vector connecting sound source and receiver run in parallel. In the near field, however, this is only true for monopole sound sources [42] and does most likely not apply for the bulk of biologically significant sound sources, let alone sound reflecting objects in the case of echolocation. These theoretical considerations, which also apply to other vocal and non-vocal fishes, in combination with the fact that the individuals observed in our study did not show the reasonably expected behavioral changes under the different experimental conditions make it highly unlikely that individuals of *A. seemanni* use their respective calls for echoacoustic orientational purposes. If echoacoustic obstacle detection with the described calls is possible at all, then only in the *immediate* vicinity of an obstacle, where changes in the hydrodynamic flow-field perceived with the lateral-line system potentially provide much more reliable information about the presence of an object (compare [43], [44]). Thus, an intra- or interspecific communicative function of the calls of *A. seemanni* would be more conceivable (compare below). In this case, the sound emitting individual would only serve the role of the sound source so that the signaling range (without the necessity of high-amplitude sound reflection) would be increased as compared to echoacoustic conditions and might become even larger if the signal perceiver belongs to the so-called hearing specialists among the fishes, as *A. seemanni* does [41].

### Possible Roles of Constant-Frequency Calls in Communication and Behavioral Experiment 3

In fishes, the behavioral contexts in which species-specific sounds occur are substantially less well studied as compared to the animals’ sound production and detection capabilities [45]. According to these authors, known circumstances of sound emission in fishes include aggression, defense, territorial advertisement, courtship, and mating. Regarding the facts that the peak energy of the vocalizations of *A. seemanni* is found in the frequency range audible to most species of fish and that about 100 members of the taxon Ariidae (sea catfishes) have recently been categorized as presumably venomous [46], an additional possible function of the presently described swim-bladder sounds arises. Comparable to the clicks of tiger moths, which bats learn to associate with unpalatable/noxious prey [47], catfish sounds could be used as a warning signal. Unlike the aposematic [48] or warning coloration of lionfish (*Pterois volitans*), which seems to advertise to predators that these fish are too venomous or spiny to be worth eating [49], acoustic displays have the clear advantage of not being affected by limited sight or light conditions. According to a recent review on human envenomations [50], punctures caused by marine catfishes via the three serrated bony stings on the dorsal and pectoral fins, which are associated with venom gland cells [46], can lead to local intense pain, oedema, erythema and paleness, and occasionally cutaneous necrosis. Catfish-venom effects, such as local inflammation, typically subside after about 6 h [50] and are produced in a wide range of vertebrates [46]. Hence, most predators should be able to both survive and remember previous negative catfish encounters.

A second possible function of the calls is to facilitate school formation. In herring, for example, sounds are highly likely involved in restoring schools from scattered individuals [51]. Since call production in *A. seemanni* did not cease in isolation (our single-fish experiments), sounds could also fulfill the function of lowering inter-individual distances in this species of fish. If so, even the necessity for directional hearing could be omitted, since the distance to the school and its direction could easily be determined by the swimming individual by comparing signal amplitudes at different points in space and time. The school could then be found or maintained by following the volume gradient in the direction of increasing call amplitude. The results of our third behavioral experiment are indicative that calls in *A. seemanni* may, indeed, play a role in schooling. In the forced choice paradigm of the Y-maze, 4 out of 5 individuals showed a clear trend (p<0.1) towards deciding for the arm from which the sound of an artificial school of conspecifics was played back. The sum of 67 out of 100 decisions for this arm is a highly significant deviation from chance level (p = 0.00044). Since (i) the Y-maze was set-up in an otherwise featureless room, (ii) no optical stimulation in the form of fish dummies was provided, and (iii) the loudspeaker was electromagnetically shielded, we are convinced that it was the acoustical information alone that caused the preference of the experimental animals for the arm with the loudspeaker. However, due to the small sample size used for behavioral experiment 3 (n = 5), further experiments will be necessary to verify our finding that individuals of *A. seemanni* show some sort of “phonotaxic” behavior towards the calls of conspecifics.

## Supporting Information

Figure S1 **Comparison of the measured parameters between the two recording environments (aquarium and pool) for fish 1.** Distributions and central tendencies of the center frequencies and relative amplitudes (using the amplitude of the belonging fundamental frequencies as a 0 dB reference) of the fundamental (h_0_) and the first three harmonics (h_1_–h_3_). Dashed lines correspond to integer multiples of the median center frequency of the fundamental. Horizontal solid lines indicate the interquartile range of the center frequencies of the fundamental and the respective harmonics, vertical solid lines the belonging interquartile range of the relative amplitudes. For the fundamental and each harmonic, the pair of solid lines crosses at the respective medians of the represented parameters.
(TIFF)

Figure S2 **Comparison of the measured parameters between the two recording environments (aquarium and pool) for fish 2.** Distributions and central tendencies of the center frequencies and relative amplitudes (using the amplitude of the belonging fundamental frequencies as a 0 dB reference) of the fundamental (h_0_) and the first three harmonics (h_1_–h_3_). Dashed lines correspond to integer multiples of the median center frequency of the fundamental. Horizontal solid lines indicate the interquartile range of the center frequencies of the fundamental and the respective harmonics, vertical solid lines the belonging interquartile range of the relative amplitudes. For the fundamental and each harmonic, the pair of solid lines crosses at the respective medians of the represented parameters. Significance code: **p<0.01; ***p<0.001 (Wilcoxon rank sum test, two-sided, confidence level = 0.95, p-values “Holm-Bonferroni”-adjusted for multiple testing).
(TIFF)

Figure S3 **Comparison of the measured parameters between the two recording environments (aquarium and pool) for fish 3.** Distributions and central tendencies of the center frequencies and relative amplitudes (using the amplitude of the belonging fundamental frequencies as a 0 dB reference) of the fundamental (h_0_) and the first three harmonics (h_1_–h_3_). Dashed lines correspond to integer multiples of the median center frequency of the fundamental. Horizontal solid lines indicate the interquartile range of the center frequencies of the fundamental and the respective harmonics, vertical solid lines the belonging interquartile range of the relative amplitudes. For the fundamental and each harmonic, the pair of solid lines crosses at the respective medians of the represented parameters. Significance code: **p<0.01 (Wilcoxon rank sum test, two-sided, confidence level = 0.95, p-values “Holm- Bonferroni”-adjusted for multiple testing).
(TIFF)

Figure S4 **Comparison of the measured parameters between the two recording environments (aquarium and pool) for fish 4.** Distributions and central tendencies of the center frequencies and relative amplitudes (using the amplitude of the belonging fundamental frequencies as a 0 dB reference) of the fundamental (h_0_) and the first three harmonics (h_1_–h_3_). Dashed lines correspond to integer multiples of the median center frequency of the fundamental. Horizontal solid lines indicate the interquartile range of the center frequencies of the fundamental and the respective harmonics, vertical solid lines the belonging interquartile range of the relative amplitudes. For the fundamental and each harmonic, the pair of solid lines crosses at the respective medians of the represented parameters.
(TIFF)

Figure S5 **Comparison of the measured parameters between the two recording environments (aquarium and pool) for fish 5.** Distributions and central tendencies of the center frequencies and relative amplitudes (using the amplitude of the belonging fundamental frequencies as a 0 dB reference) of the fundamental (h_0_) and the first three harmonics (h_1_–h_3_). Dashed lines correspond to integer multiples of the median center frequency of the fundamental. Horizontal solid lines indicate the interquartile range of the center frequencies of the fundamental and the respective harmonics, vertical solid lines the belonging interquartile range of the relative amplitudes. For the fundamental and each harmonic, the pair of solid lines crosses at the respective medians of the represented parameters.
(TIFF)

Figure S6 **Comparison of the measured parameters between the two recording environments (aquarium and pool) for fish 6.** Distributions and central tendencies of the center frequencies and relative amplitudes (using the amplitude of the belonging fundamental frequencies as a 0 dB reference) of the fundamental (h_0_) and the first three harmonics (h_1_–h_3_). Dashed lines correspond to integer multiples of the median center frequency of the fundamental. Horizontal solid lines indicate the interquartile range of the center frequencies of the fundamental and the respective harmonics, vertical solid lines the belonging interquartile range of the relative amplitudes. For the fundamental and each harmonic, the pair of solid lines crosses at the respective medians of the represented parameters.
(TIFF)

Figure S7 **Comparison of the measured parameters between the two recording environments (aquarium and pool) for fish 7.** Distributions and central tendencies of the center frequencies and relative amplitudes (using the amplitude of the belonging fundamental frequencies as a 0 dB reference) of the fundamental (h_0_) and the first three harmonics (h_1_–h_3_). Dashed lines correspond to integer multiples of the median center frequency of the fundamental. Horizontal solid lines indicate the interquartile range of the center frequencies of the fundamental and the respective harmonics, vertical solid lines the belonging interquartile range of the relative amplitudes. For the fundamental and each harmonic, the pair of solid lines crosses at the respective medians of the represented parameters.
(TIFF)
