# Supplementary figures and images for: Structure and Possible Functions of Constant-Frequency Calls in Ariopsis seemanni (Osteichthyes, Ariidae)
Source: PLoS One. 2013 May 31;8(5):e64864. doi: 10.1371/journal.pone.0064864 (PMC3669340; doi:10.1371/journal.pone.0064864)

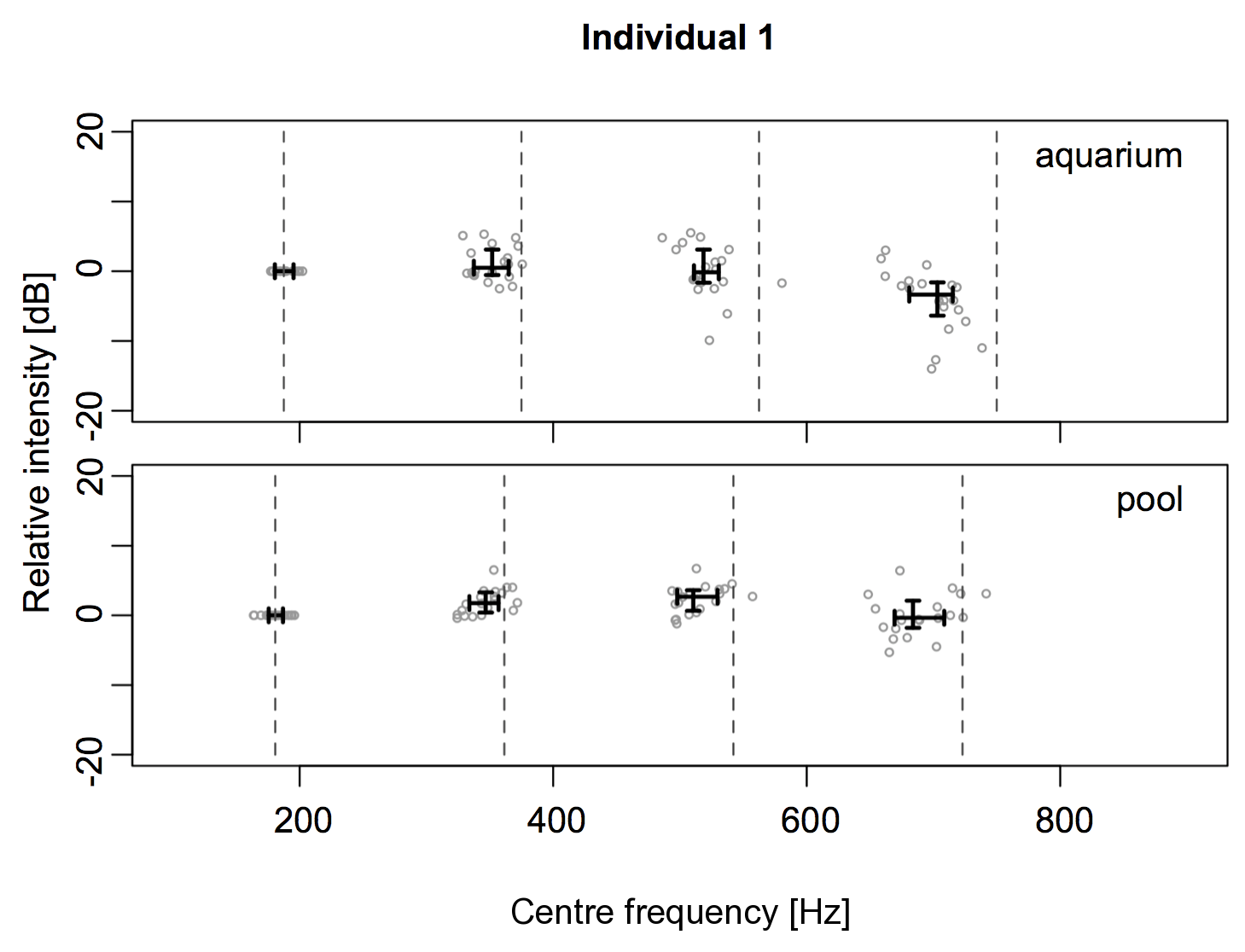

Supplement: Figure S1 — Comparison of the measured parameters between the two recording environments (aquarium and pool) for fish 1. Distributions and central tendencies of the center frequencies and relative amplitudes (using the amplitude of the belonging fundamental frequencies as a 0 dB reference) of the fundamental (h0) and the first three harmonics (h1–h3). Dashed lines correspond to integer multiples of the median center frequency of the fundamental. Horizontal solid lines indicate the interquartile range of the center frequencies of the fundamental and the respective harmonics, vertical solid lines the belonging interquartile range of the relative amplitudes. For the fundamental and each harmonic, the pair of solid lines crosses at the respective medians of the represented parameters. (TIFF) [file pone.0064864.s001.tiff]

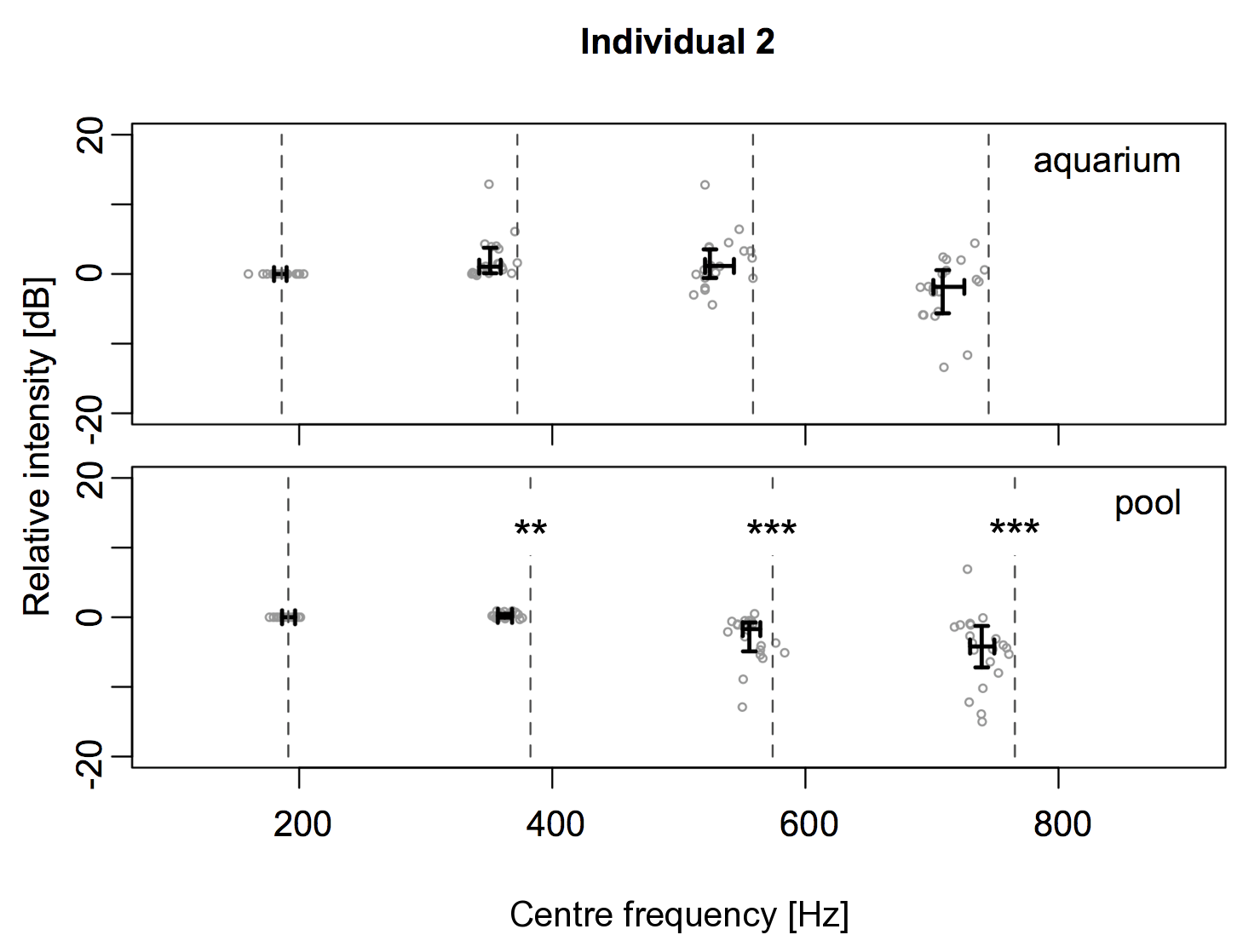

Supplement: Figure S2 — Comparison of the measured parameters between the two recording environments (aquarium and pool) for fish 2. Distributions and central tendencies of the center frequencies and relative amplitudes (using the amplitude of the belonging fundamental frequencies as a 0 dB reference) of the fundamental (h0) and the first three harmonics (h1–h3). Dashed lines correspond to integer multiples of the median center frequency of the fundamental. Horizontal solid lines indicate the interquartile range of the center frequencies of the fundamental and the respective harmonics, vertical solid lines the belonging interquartile range of the relative amplitudes. For the fundamental and each harmonic, the pair of solid lines crosses at the respective medians of the represented parameters. Significance code: **p<0.01; ***p<0.001 (Wilcoxon rank sum test, two-sided, confidence level = 0.95, p-values “Holm-Bonferroni”-adjusted for multiple testing). (TIFF) [file pone.0064864.s002.tiff]

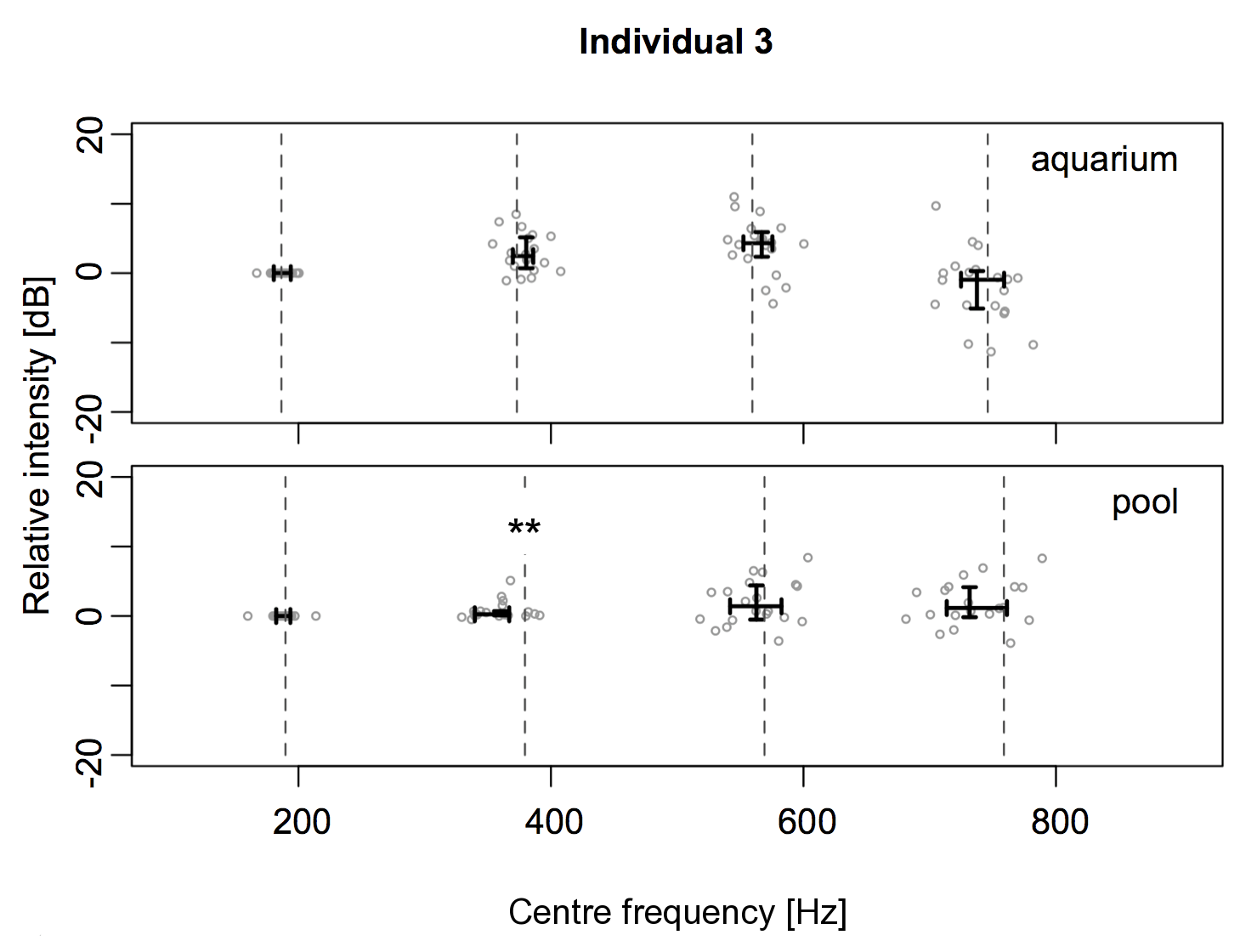

Supplement: Figure S3 — Comparison of the measured parameters between the two recording environments (aquarium and pool) for fish 3. Distributions and central tendencies of the center frequencies and relative amplitudes (using the amplitude of the belonging fundamental frequencies as a 0 dB reference) of the fundamental (h0) and the first three harmonics (h1–h3). Dashed lines correspond to integer multiples of the median center frequency of the fundamental. Horizontal solid lines indicate the interquartile range of the center frequencies of the fundamental and the respective harmonics, vertical solid lines the belonging interquartile range of the relative amplitudes. For the fundamental and each harmonic, the pair of solid lines crosses at the respective medians of the represented parameters. Significance code: **p<0.01 (Wilcoxon rank sum test, two-sided, confidence level = 0.95, p-values “Holm- Bonferroni”-adjusted for multiple testing). (TIFF) [file pone.0064864.s003.tiff]

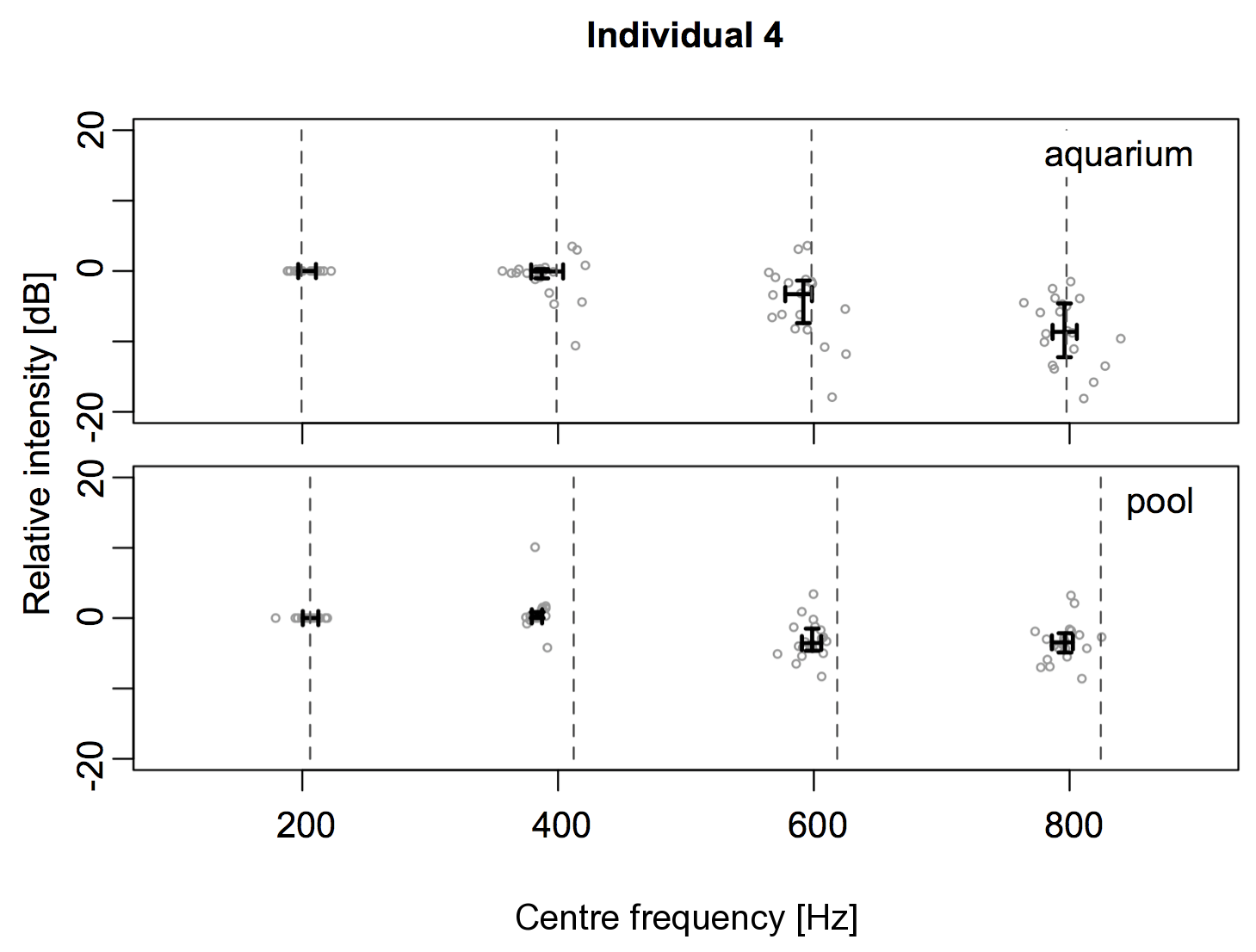

Supplement: Figure S4 — Comparison of the measured parameters between the two recording environments (aquarium and pool) for fish 4. Distributions and central tendencies of the center frequencies and relative amplitudes (using the amplitude of the belonging fundamental frequencies as a 0 dB reference) of the fundamental (h0) and the first three harmonics (h1–h3). Dashed lines correspond to integer multiples of the median center frequency of the fundamental. Horizontal solid lines indicate the interquartile range of the center frequencies of the fundamental and the respective harmonics, vertical solid lines the belonging interquartile range of the relative amplitudes. For the fundamental and each harmonic, the pair of solid lines crosses at the respective medians of the represented parameters. (TIFF) [file pone.0064864.s004.tiff]

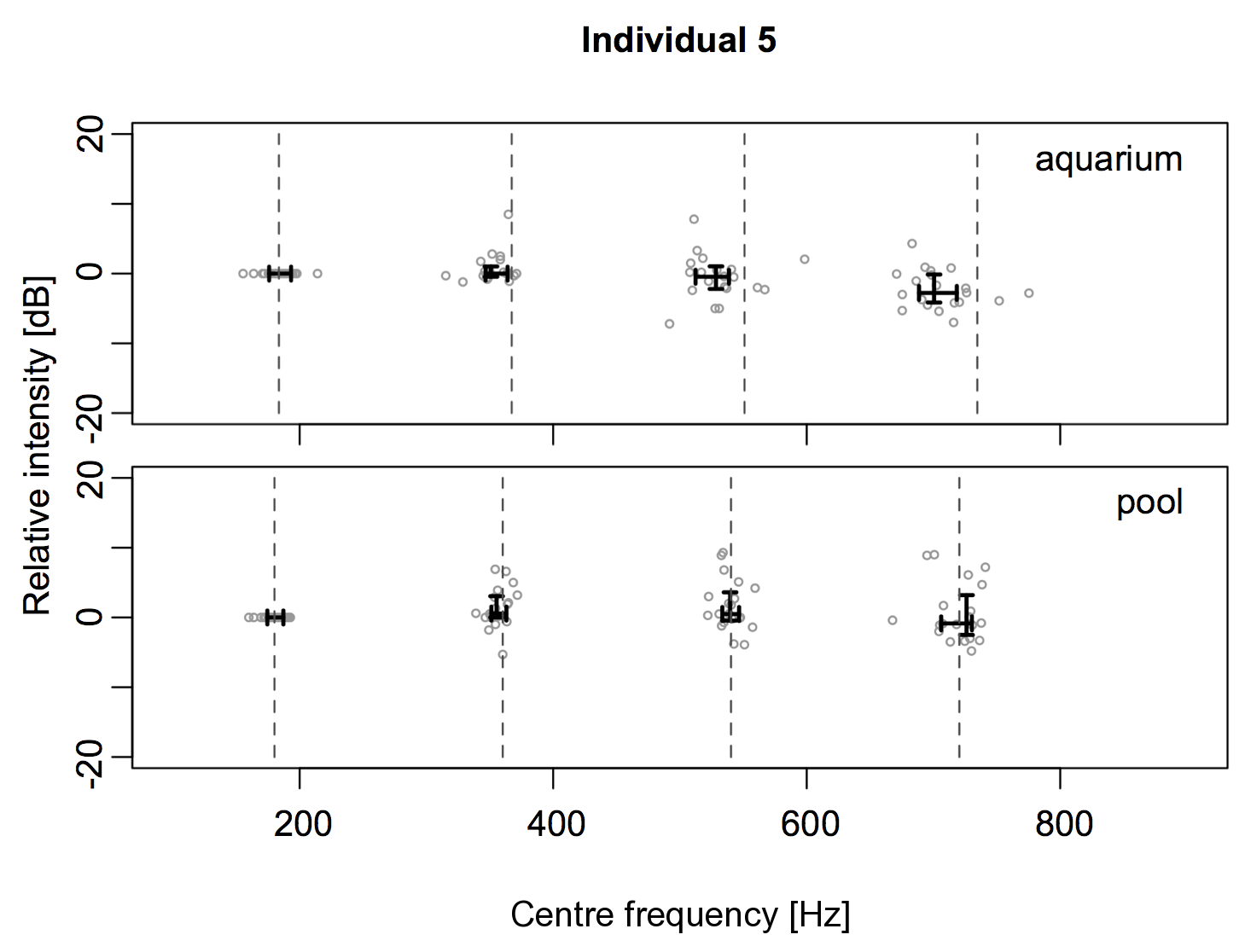

Supplement: Figure S5 — Comparison of the measured parameters between the two recording environments (aquarium and pool) for fish 5. Distributions and central tendencies of the center frequencies and relative amplitudes (using the amplitude of the belonging fundamental frequencies as a 0 dB reference) of the fundamental (h0) and the first three harmonics (h1–h3). Dashed lines correspond to integer multiples of the median center frequency of the fundamental. Horizontal solid lines indicate the interquartile range of the center frequencies of the fundamental and the respective harmonics, vertical solid lines the belonging interquartile range of the relative amplitudes. For the fundamental and each harmonic, the pair of solid lines crosses at the respective medians of the represented parameters. (TIFF) [file pone.0064864.s005.tiff]

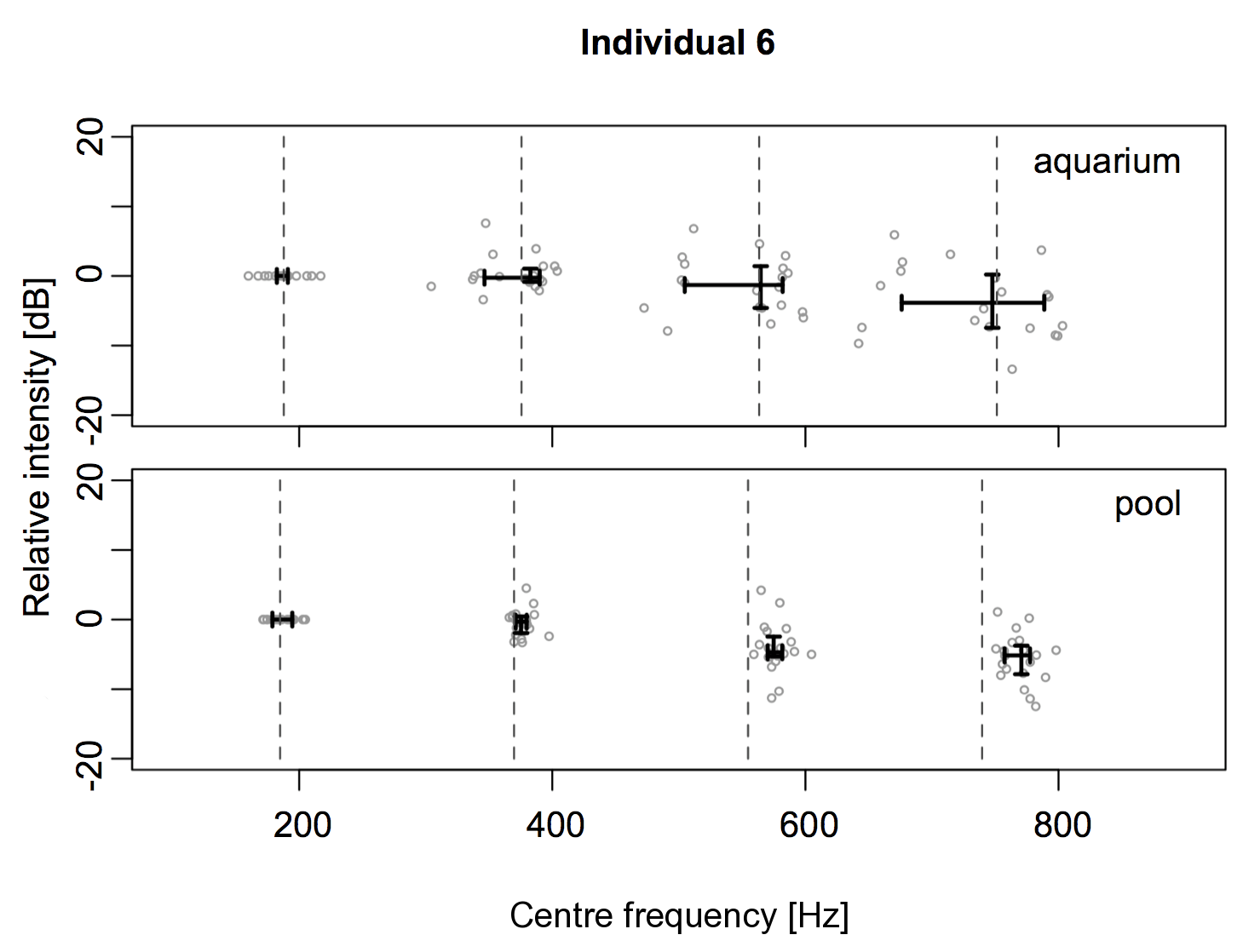

Supplement: Figure S6 — Comparison of the measured parameters between the two recording environments (aquarium and pool) for fish 6. Distributions and central tendencies of the center frequencies and relative amplitudes (using the amplitude of the belonging fundamental frequencies as a 0 dB reference) of the fundamental (h0) and the first three harmonics (h1–h3). Dashed lines correspond to integer multiples of the median center frequency of the fundamental. Horizontal solid lines indicate the interquartile range of the center frequencies of the fundamental and the respective harmonics, vertical solid lines the belonging interquartile range of the relative amplitudes. For the fundamental and each harmonic, the pair of solid lines crosses at the respective medians of the represented parameters. (TIFF) [file pone.0064864.s006.tiff]

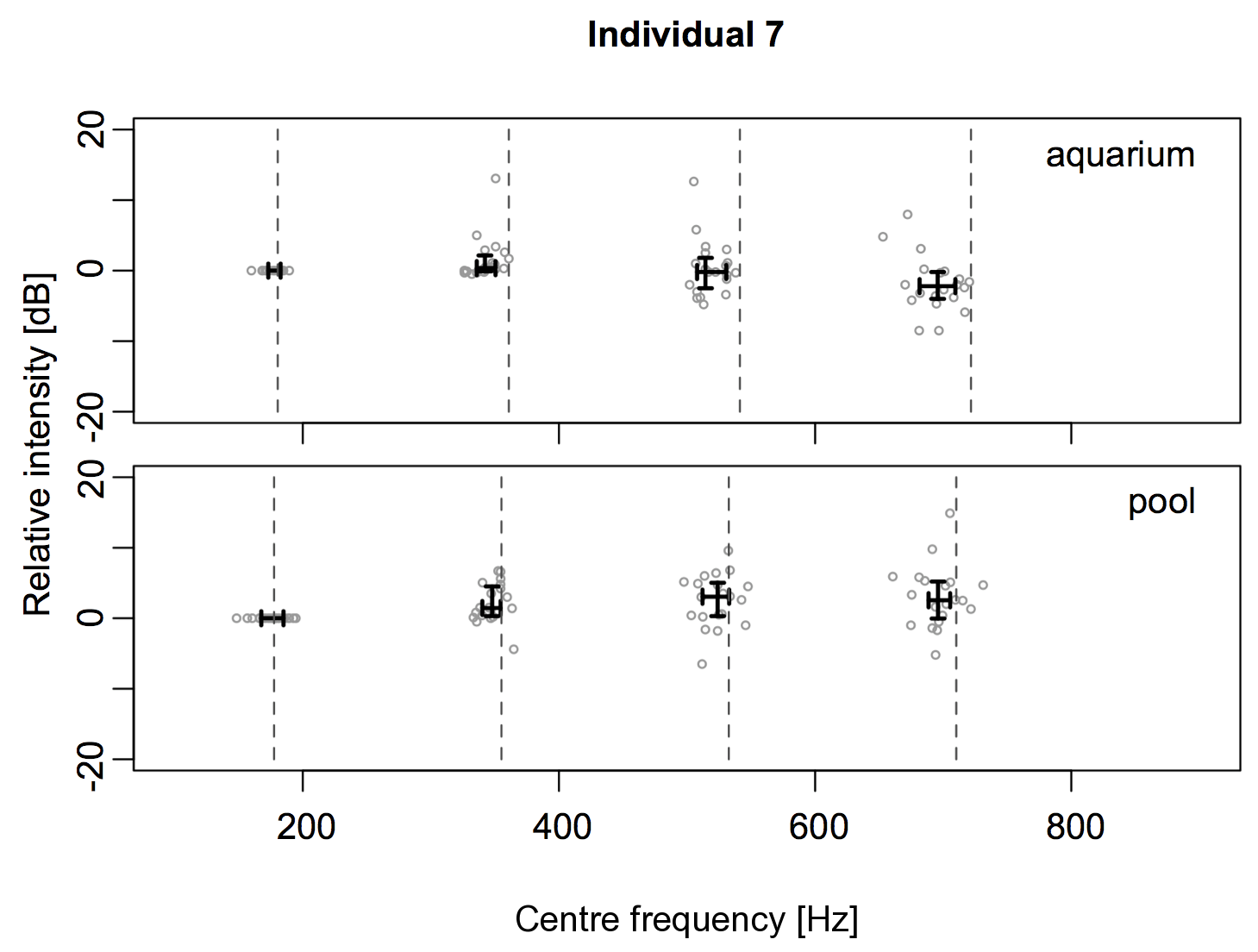

Supplement: Figure S7 — Comparison of the measured parameters between the two recording environments (aquarium and pool) for fish 7. Distributions and central tendencies of the center frequencies and relative amplitudes (using the amplitude of the belonging fundamental frequencies as a 0 dB reference) of the fundamental (h0) and the first three harmonics (h1–h3). Dashed lines correspond to integer multiples of the median center frequency of the fundamental. Horizontal solid lines indicate the interquartile range of the center frequencies of the fundamental and the respective harmonics, vertical solid lines the belonging interquartile range of the relative amplitudes. For the fundamental and each harmonic, the pair of solid lines crosses at the respective medians of the represented parameters. (TIFF) [file pone.0064864.s007.tiff]
